# Iron oxide nanoparticles may damage to the neural tissue through iron accumulation, oxidative stress, and protein aggregation

**DOI:** 10.1186/s12868-017-0369-9

**Published:** 2017-06-26

**Authors:** Zahra Yarjanli, Kamran Ghaedi, Abolghasem Esmaeili, Soheila Rahgozar, Ali Zarrabi

**Affiliations:** 1 Department of Biology, Faculty of Sciences, University of Isfahan, Hezar Jerib Ave., Azadi Square, Isfahan, 81746-73441 Iran; 2 Department of Biotechnology, Faculty of Advanced Sciences and Technologies, University of Isfahan, Hezar Jerib Ave., Azadi Square, Isfahan, 81746-73441 Iran

**Keywords:** Iron, Iron accumulation, Iron oxide nanoparticles, Neurodegenerative diseases, Oxidative stress, Protein aggregation

## Abstract

**Background:**

In the recent decade, iron oxide nanoparticles (IONPs) have been proposed for several applications in the central nervous system (CNS), including targeting amyloid beta (Aβ) in the arteries, inhibiting the microglial cells, delivering drugs, and increasing contrast in magnetic resonance imaging. Conversely, a notable number of studies have reported the role of iron in neurodegenerative diseases. Therefore, this study has reviewed the recent studies to determine whether IONPs iron can threaten the cellular viability same as iron.

**Results:**

Iron contributes in Fenton’s reaction and produces reactive oxygen species (ROS). ROS cause to damage the macromolecules and organelles of the cell via oxidative stress. Iron accumulation and oxidative stress are able to aggregate some proteins, including Aβ and α-synuclein, which play a critical role in Alzheimer’s and Parkinson’s diseases, respectively. Iron accumulation, oxidative stress, and protein aggregation make a positive feedback loop, which can be toxic for the cell. The release of iron ions from IONPs may result in iron accumulation in the targeted tissue, and thus, activate the positive feedback loop. However, the levels of IONPs induced toxicity depend on the size, concentration, surface charge, and the type of coating and functional groups of IONPs.

**Conclusion:**

IONPs depending on their properties can lead to iron accumulation, oxidative stress and protein aggregation in the neural cells. Therefore, in order to apply IONPs in the CNS, the consideration of IONPs properties is crucial.

## Background

In the recent years, iron oxide nanoparticles (IONPs) have been under special interests, due to their ability to target a certain site within the body under an external magnetic field [1]. Also, IONPs can surpass the blood brain barrier (BBB) [2]. Therefore, they have been proposed for a variety of applications in the central nervous system (CNS), including targeting amyloid beta (Aβ) in the arteries [3], inhibiting the microglial cells [4], delivering a drug [5], and increasing contrast in magnetic resonance imaging (MRI) [6].

IONPs are composed of two components: a nucleus of iron oxide and a hydrophilic coating sheet such as dextran or poly ethylene glycol [2]. Magnetite (Fe_3_O_4_) and maghemite (γ-Fe_2_O_3_)—two compounds of iron oxide—are frequently used in biomedical applications. The physical properties of both compounds are very similar; Fe^2+^ ions in the magnetite have a higher ionic radius than Fe^3+^ ions in the maghemite [7]. The hydrodynamic radius and surface charge of nanoparticle (NP) determines the protection period of NP in circulation, the accessibility of tissues, opsonization, and its absorption by the cell [2].

Upon cell absorption, IONPs, locate in the acidic medium of lysosomes, where IONPs are metabolized and produce free iron ions into the cells [8]. In addition, the direction of IONPs using an external magnetic field toward a specific tissue [1], lead to iron accumulation in the targeted tissue [9]. Given to the widespread use of IONPs on one side and toxicity of iron in neurodegenerative disorders at the other side, this study has reviewed the recent studies to determine whether IONPs, as well as iron, can influence the cellular activities and metabolism.

## Methods

We carried out a data mining through a deep manual research in PubMed using a combination of several keywords, including the toxic effects of iron oxide nanoparticle, iron accumulation, and neurodegenerative diseases. In our data mining, the most of included research and review articles were published in PubMed after 2002.

## Results

### Transportation and hemostasis of iron in the cell

Iron is a metal ion at the body which plays a critical role in various physiological functions, including DNA synthesis, mitochondrial respiration, and oxygen transport [10]. Iron in the food sources primarily is absorbed via endocytosis—mediated by divalent metal transporter1 (DMT1)—in the duodenum, and is transported by transferrin in blood. Transferrin, bonds its receptor (TfR) on the surface of the target cells and enters into the cell by receptor mediated-endocytosis [11]. The acidic medium of endosome causes to release iron from transferrin. The resulted iron introduces to the cytoplasm by the DMT1 transporter. Iron can be transported to mitochondria and contribute in the synthesis of heme or sulfur-iron cluster and/or may remain in the cytoplasm and be stored by ferritin [12].

Under normal conditions, iron binds ferritin in the redox-inactive Fe^3+^ state and a small quantity of redox-active Fe^2+^ irons are needed to contribute to the cellular metabolism. Therefore, all mechanisms involving transportation and homeostasis of iron must be strongly regulated to prevent excess iron from cytotoxic reactions [13]. More regulation of iron homeostasis in the cell is carried out by iron response protein (IRP)/iron response element (IRE) system. If IRE is located in 5′-UTR of target genes mRNA, in presence if iron, IRP cannot connect to IRE, thus mRNA would be translated and protein can be synthesized; such as ferritin. However, when IRE is in 3′-UTR of mRNA, in presence of iron, mRNA would be degraded by nucleases, and thus related protein cannot be synthesized such as TfR and DMT [12].

Given to the role of iron in physiologic functions, any disruption in the regulation of iron transportation or homeostasis results to increase or decrease the amount of iron in the cell and can affect physiologic functions.

### The role of iron in oxidative stress

Iron is a transition metal and able to give and receive an electron. Hence, additional content of iron can be cytotoxic [14]. Iron (Fe^2+^) in natural and biological environments reacts with hydrogen peroxide (H_2_O_2_) and produces OH· radical, which reported by H.J.H. Fenton over one hundred years ago. At the present time, Fenton’s reaction is accounted for one of the most effective procedures for the oxidation of organic pollutants [15]. Therefore, Fe^2+^ in the cytoplasm can participate in the Fenton’s reaction (Eq. 1) and lead to the generation of reactive oxygen species (ROS).

1 $${\text{Fe}}^{2 + } + {\text{H}}_{2} {\text{O}}_{2}\rightarrow {\text{Fe}}^{3 + } + {\text{OH}}^{ - } + {\text{OH}}^{ \cdot }$$

Excess iron in the cytoplasm is arrested by ferritin. The free amount of iron can exert toxic effects on the cell. Núñez et al. in their review acknowledged that ascorbate and reduced glutathione (GSH) in the cell act as reluctant to regenerate Fe^2+^. The intracellular amount of Fe^2+^, in presence of both oxygen and GSH, is also able to produce ROS [16]. Hence, production of ROS by iron accumulation is inevitable in the cell. Basically, ROS regulate the normal activities of the cell, but an abnormal increase of ROS levels may damage the cell [17]. ROS increase the permeability of the outer mitochondrial membrane, damage lysosomal membrane [18], and trigger release of iron from these organelles. Thus, ROS may enhance iron accumulation in the cytosol. ROS produced by iron also reacts with the cell membrane and causes lipid peroxidation. During this reaction, ROS generates toxic aldehyde products, including malondialdehyde and 4-hydroxylnonenal [19]. These toxic components react with proteins to produce carbonyl functions, which damage the proteins. The damaged, misfolded proteins cannot be recognized by ubiquitin/proteasome system, and they aggregate within the cytoplasm as seen in neurodegenerative diseases [12]. Furthermore, ROS can modify DNA by degradation of bases, break of DNA chain, mutations, modification of purine, pyrimidine or sugar-bound, deletions or translocations, and cross-linking with proteins. These modifications may have relation with aging, cancer, and neurodegenerative diseases [20]. ROS can initiate cellular injuries by modifying lipids, proteins, and DNA or lead to generate secondary ROS and finally cell death [21]. Accordingly, excess iron can produce ROS via Fenton’s reaction, causes oxidative stress, and finally cell damage.

### The role of iron in the CNS

Iron has an essential role in many metabolic processes in the CNS, including oxidative phosphorylation, myelin synthesis, neurotransmitter production such as dopamine and serotonin, and nitric oxide metabolism. Iron also acts as a cofactor for tyrosine hydroxylase [12]. This enzyme has a necessary role in dopamine synthesis, and the inhibition of its activity can damage to the function and viability of neural cell [22]. Thus, iron is an essential factor for proper function of neurons. CNS is more sensitive to oxidative stress, because enzymes, which are responsible for removing free radicles, including catalase, superoxide dismutase, and glutathione peroxidase [21], have less activity in the brain [23]. Due to the ability of iron for producing ROS, excess iron within the brain can be more dangerous than other tissues. In addition, increased iron level in the brain suppresses occludin expression. Given the role of occluding, a protein of tight junction, in the BBB, reducing its expression may disturb the function of BBB and thus damage the brain [19]. Although, iron is essential for many metabolic processes, excess iron can be toxic to the brain. Therefore, any change in iron level may be dangerous for the health of human.

### The role of iron in Parkinson disease

There are some inherited neurodegenerative diseases, including neurodegeneration with brain iron accumulation [1], Aceruloplasminemia and Neuroferritinopathy, which associated with iron accumulation in the specific areas—such as *Substantia nigra* (Sn)—of the brain. Patients with these diseases show symptoms of Parkinson. Also, animal models of Parkinson, created in the laboratory by treating with 6-OHDA, indicate an increased iron level in the Sn. These facts demonstrate a relationship between iron accumulation and neurodegenerative diseases [24]. Parallel with iron accumulation, it has been observed a reduction of Tau and amyloid precursor protein (APP) levels within the brain of Parkinson’s patients. Tau and APP are involved in iron export from the cell; APP stabilizes ferroportin on the cell membrane and Tau interacts with APP to facilitate iron export [25]. This proposes a disruption in regulating of iron level within the brain, can be a factor contributing to the accumulation of iron in PD.

### Iron accumulation and GSH, dopamine, and neuromelanin in Sn

GSH is the most abundant antioxidant in all compartments of the cell [20]. Nu´n˜ez et al. proposed there is a positive feedback-loop between iron accumulation, low GSH, and oxidative stress. Iron accumulation causes to decrease the level of GSH and induction of oxidative stress. Furthermore, the low level of GSH results in increasing the level of TfR, which enters iron into the cell, and aids iron accumulation. Chemicals which inhibit complex1 in mitochondria such as 6-hydroxydopamine (6-OHDA) or inflammatory factors activate this loop [16].

Dopamine is a neurotransmitter secreted by dopaminergic neurons in Sn. Recently, Hare et al. reviewed iron-dopamine couple and proposed that the interaction of iron and dopamine can generate neurotoxic byproducts, including 6-hydroxydopamine quinone (6-OHDA-Q), 6-OHDA, tetrahydroisoquinoline, and H_2_O_2_. These products activate a loop between iron accumulation, low GSH, and oxidative stress. Therefore, dopamine intensifies toxicity of iron accumulation and makes a more toxic situation for neurons in the Sn [25].

In dopaminergic cells, ferritin level is lower than other regions throughout the brain, neuromelanin pigment—a dopamine product-stores free iron ions. Iron first connects to high affinity-iron binding sites and reduces the toxicity of iron, but after the occupation of these sites, iron attaches to high affinity-iron binding sites and is stored in Fe^2+^ state [16]. Then, when iron is higher than of a threshold, neuromelanin helps to keep redox-active iron in the cell; and neuromelanin, itself is considered as a destructive factor.

Given above content, we propose when iron accumulation occurs, low GSH, dopamine, and neuromelanin in the Sn help to more accumulate iron and contribute to more generation of oxidative stress.

### The role of iron in α-sinculein aggregation

Abnormal deposition of proteins into the brain is a characteristic feature of several neurodegenerative disorders and age-related diseases, including Parkinson’s disease. However, the composition and location of protein aggregations are different among diseases [26–30]. There are protein aggregates, known as lewy bodies, containing α-sinculein (αS)—non-amyloid component of APP—and ubiquitin, in the midbrain of Parkinson’s patients [25, 26]. The observation of iron accumulation within the brain of Parkinson’s patients suggests a link between iron and aggregation of αS [31]. Also, the existence of IRE sequences in 5′UTR of αS mRNA indicates that αS has a role in iron hemostasis [11]. The overexpression of αS results iron accumulation in neural cells. Hare et al. proposed that αS in the aggregated form can store iron, similar to neuromelanin. Mutually, the free iron and iron-dopamine complex available in the Sn may interact with aS and produce a form of αS, which cannot be distinguished by ubiquitin/proteasome system and therefore, causes αS aggregation [25]. αS in aggregates can produce H_2_O_2_ which in the presence of iron generates OH radicals [24]. Together with, αS aggregation, iron accumulation and oxidative stress make a positive feedback-loop in which iron accumulation results in oxidative stress and αS aggregation. αS aggregation leads to more iron accumulation and further oxidative stress.

### The role of iron in Alzheimer disease

Alzheimer disease is a neurodegenerative disorder that its prevalence is increasing in the world. The main features of Alzheimer are a loss of synapses, increased number of senile plaques (containing Aβ) outside the neuron, and promoted fibrillar aggregation of protein tau inside the neuron [32]. There is a relationship between the aggregation of Tau in the hippocampus and declined cognition in AD. The effect of Iron on protein aggregation was explained earlier. Iron accumulation and protein aggregation, both result in extensive oxidative stress, which disturbs synaptic function [16]. Furthermore, iron accumulation leads to loss of dendritic spines [19], which contributes to a number of neuropsychiatric disorders [33]. Thus we propose that iron accumulation has a role in cognitive suppression in AD.

### The role of iron in Aβ aggregation

Some studies have reported that iron accumulates within the brain of Alzheimer’s patients, and also proposed the using iron chelators to improve this disease [10, 31, 34]. Intracellular free iron may affect the expression of some proteins such as amyloid precursor protein. Salvador et al. showed a correlation between the high concentration of iron and oxidative stress and altered Aβ concentration in both soluble and deposited forms [35]. In physiological-like condition, iron can bind to Aβ and deposit it as amyloid aggregates [34]. Also, Aβ in the aggregates can reduce Fe^3+^ to Fe^2+^ [36], which by contributing to Fenton’s reaction, may lead to further ROS, and ROS finally intensifies aggregation of Aβ [19]. Researchers have claimed that by removing the iron ions from senile plaques, they could reduce the toxicity of the plaques and enhance the solubility of amyloid [34]. This explanation shows a positive feedback-loop between iron accumulation, oxidative stress, and protein aggregation. It seems this loop is an effective factor in worsening of the situation in neurodegenerative diseases and using iron chelators may prevent the progress of these diseases.

### The role of iron in the cell death

Recently, a type of cellular death on the basis of iron ions accumulation, called as ferroptosis, has been defined. This type of cellular death has morphological, biochemical and genetic properties different from apoptosis, necrosis and autophagy. In this situation, mitochondria become smaller than normal and the density of their membranes increases. Dixton et al. stated that iron accumulation causes to increase the cytotoxic lipid ROS in the cell; and indicated that iron chelators, for instance deferoxamine, may prevents this death by removing the excess iron ions [37]. Hare et al. proposed iron interacted with dopamine and generated 6-OHQD, which covalently interacts with mitochondrial glutathione peroxidase 4 (GPx4) which leads to ferroptotic cell death [25]. Hence, ferroptosis probably is the cause of death in dopaminergic neurons of Sn, which lead to a deficiency of dopamine in the striatum, which in turn, cause to develop Parkinson symptoms [27].

Mantzaris et al. reported that iron level increased in H_2_O_2_-induced cell death. By inducing oxidative stress, H_2_O_2_ resulted in damaged lysosome and release of iron ions into the cytoplasm. Thus intracellular iron pool increases. Also, they indicated increased transcription of ferritin and loss of transferrin receptor on the surface of cell membrane. These results showed mechanistic regulating iron hemostasis was activated in H_2_O_2_-induced cell death [38].

A recent study has reported symptoms of apoptotic cell death, including reduced expression of Bcl-2 and increased expression of Box, in the cells of Rat’s brain with a high iron diet for four months. Elevated iron has induced apoptosis through oxidative stress. However, deferoxamine could decline iron accumulation and its toxic effects [19]. As we have reviewed recently, iron accumulation in the cytoplasm can damage to mitochondria, and thus lead to disruption in ATP synthesis and Ca^2+^ buffering. Iron, upon entrance to the mitochondria, may be used in heme and Fe-S cluster synthesis or stored by mitochondrial ferritin. Excess iron ions in the mitochondria can open mitochondrial permeability transition pore; cause the release of Ca^2+^ and cytochrome C into the cytoplasm; and result in apoptosis activation [11]. Accordingly, the excess contents of iron can lead to cell death. However, more detailed studies are required to unravel the iron effect on the various cell types.

### The role of IONPs in neurodegenerative diseases

In order to review recent studies about the role of IONPs in neurodegenerative diseases, we provided two tables from recent in vitro and in vivo studies (Tables 1 and 2). There are conflicting data related to the effects of various IONPs in different cells and organisms. Imam et al. indicated IONPs led to a reduction of the dopaminergic neurons in the striatum of rats [39], which can trigger PD. Zhang et al. reversely showed IONPs had neuroprotective effects and diminished the toxic effects of MPP (an inducer of Parkinson) in PD model-cells. In addition, dietary IONPs could protect AD-model *Drosophila* against neurodegeneration; and delayed aging [40]. For analyzing the role of IONPs in neurodegenerative disorders, it seems necessary to reply this question; whether IONPs can activate the positive feedback loop between iron accumulation, oxidative stress, and protein aggregation. If these NPs, or the iron ions released from these particles, are able to do this, IONPs can be effective in promoting the neurodegenerative diseases. So, we briefly discuss the roles of IONPs in iron accumulation, oxidative stress, and protein aggregation.

**Table 1:** The in vitro effects of IONPs

Cell type	NP type	Size (nm)	Concentration (μg mL^−1^)	Coating	Functional group	Explantations
Murine macrophage cell line (J774)	Fe_2_O_3_	30	25–500	Tween 80	Hydroxyl	Dose- and time-dependent reduction of viability, cell membrane damage, and induction of apoptosis by ROS [17]
Chick cortical neurons	Fe_3_O_4_	∼10	2655, 5310	Aminosilane	Amine	PEA, compared with others coatings, strongly declined metabolic activity and cell viability and destroyed cell membrane [45].
				Dextran	Hydroxyl	
				PEA	Amine	
Porcine aortic endothelial cells	Fe_3_O_4_	5 or 30	500	None (Bare)	Hydroxyl	Significant increasing of cell elongation and cell death were seen by bare NPs. Bare 30-NPs incited ROS formation; but coated 30-NPs and bare 5-NPs didn’t induce significant ROS formation. [50].
				Dextran	Hydroxyl	
				PEG	Hydroxyl	
Human dermal fibroblast	Fe_3_O_4_	10	0–1000	None (Bare)	Hydroxyl	Coating of NPs by TEOS-APTMS and APTMS intensified toxicity and led to a dose-dependent decreased viability, membrane damage, and declined the stability of DNA [46].
		100–150	0–1000	SiO_2_ and TEOS	Hydroxyl	
		100–150	0–1000	SiO_2_, TEOS, and APTMS	Amine	
		10	0–1000	APTMS	Amine	
Human fibrosarcoma cells	Fe_3_O_4_	10	0–800	None (Bare)	Hydroxyl	Membrane damage and decreased the stability of DNA [46]
Rat pheochromocytoma cells (PC12)	Fe_2_O_3_	∼36	25–200	APTS	Amine	Increased ROS, reduced GSH, and induced apoptosis [55]
Human breast cancer cell line (MCF-7)	Fe_3_O_4_	∼11	50–200	None (Bare)	Hydroxyl	Dose-dependent reduction of viability [66]
Human neuroblastoma cell line (SH-SY5Y)	Fe_2_O_3_	∼10	2.5–10	None (Bare)	Hydroxyl	Decreased dopamine levels, induction of oxidative stress, and reducing of cell proliferation [39]
		∼30	2.5–10	Oleic acid and PEG	Carboxyl	
Rat brain microvessel endothelial cells	Fe_2_O_3_	∼10	1, 10, and100	None (bare)	Hydroxyl	Significant increase in ROS level by 10 nm-NPs. Damage to the membrane by both NPs [39].
		∼30	1–100	Oleic acid and PEG	Carboxyl	
Human hepatocyte carcinoma cell line (Hep G-2)	Hollow sphere Fe_2_O_3_	200	25–200	Carbon particles	Hydroxyl and carbonyl	Dose-dependent diminished viability [56]
Mouse Fibroblastic Cell Line (L929)	Fe_3_O_4_	20	100	None (bare)	Hydroxyl	Decreased toxicity of H_2_O_2_ [40]
Rat pheochromocytoma cells (PC12)	Fe_3_O_4_	20	100	None (bare)	Hydroxyl	Decreased toxicity of MPP^+^ [40]
Human Ovarian Cancer Cell Line (Skov-3)	Fe_3_O_4_	9.2	120–240	PEG, PEI and Folic acid	Carboxyl and hydroxyl	Non-tixic [52]
Human blood cells	Fe_3_O_4_	<20	10–1000	Oleylamine	Amine	Increased oxidative stress, dose-dependent DNA damage [67]
Mouse embryonic fibroblasts (NIH3T3)	Fe_3_O_4_	15–20	0.032 and 0.065	Oleate	Carboxyl	Dose and time dependent reduced viability [51]
Mouse embryonic neural stem cells	Fe_2_O_3_	∼100^a^	20–200	bare	Hydroxyl	Severe diminished GSH, declined ROS, increased mitochondrial potential, long-term depolarization of cell membrane, and DNA damage [58]
		∼100^a^	20–200	d-mannose	Hydroxyl	
		100–150^a^	20–200	poly-l-lysine	Amine	

The studies indicate Fe^3+^ is more toxic than Fe^2+^. Amine functional group often increases cytotoxicity. The toxicity of NPs is further dose- and time-dependent

*APTMS* aminopropyltrimethoxysilane, *APTS* aminopropyltriethoxysilane, *PEA* poly-(dimethylamine-coepichlorhydrin-co-ethylendiamine), *PEG* polyethylene glycol, *PEI* polyethylenimine, *TEOS* Tetraethylorthosilicate

^a^Aggregate diameter

**Table 2:** The in vivo effects of IONPs

Organism (method)	NP type	Size (nm)	Concentration	Coating	Functional group	Explantations
Rat (Intratracheal instillation)	Fe_3_O_4_	<50	1 and 5 mg kg^−1^	None (bare)	Hydroxyl	Reduction of body weight [68]
Rat (Oral)	Fe_2_O_3_	30	30, 300, and 1000 mg kg^−1^ day^−1^	None (bare)	Hydroxyl	Significant inhibition of Na^+^–K^+^, Mg^2+^, and Ca^2+^ ATPases in brain, reduction of body weight in high dose, and reduced activity of acetyl choline esterase in the brain in high dose [64]
Rat (Intranasal)	Fe_2_O_3_	36	20 µg µL^−1^	APTS	Amine	Significant increased oxidative stress and delay in removing of NPs from stratum and hippocampus [55]
Rat (Intravascular injection)	Fe_2_O_3_	10	50 mg kg^−1^	None (bare)	Hydroxyl	Significantly decreased dopamine in striatum and dopaminergic neurons damage [39]
Rat# (Injection into stratum)	Fe_3_O_4_	6.5 ± 3.0	3 µg NP 2 µL^−1^ in a CSF	None (bare)	Hydroxyl	Increased body weight and reduction of oxidative stress in stratum [60]
Mouse (Intravascular injection)	Fe_3_O_4_	5	0.4, 2, and 10 µg kg^−1^	PEG	Hydroxyl	Induction of oxidative stress and DNA damage in heart [69]
*Drosophila* (Exposure)	Fe_3_O_4_	20	200 μg mL^−1^	None (bare)	Hydroxyl	Significant reduced ROS levels, enhanced climbing ability, and increased longevity in six-week-old flies [40]
Green algae (Raphidocelis subcapitata) (Exposure)	Fe_2_O_3_	33.3*	1–100 mg L^−1^	None (bare)	Hydroxyl	Inhibition of growth by coated and uncoated NPs after 72 h [70]
		50.4*	1–100 mg L^−1^	dimercaptosuccinic acid	Thiol and Carboxyl	
Duckweed (Lemna minor) (Exposure)	Fe_2_O_3_	33.3*	1–100 mg L^−1^	None (bare)	Hydroxyl	Unaffected by NPs in this range of doses [70]
		50.4*	1–100 mg L^−1^	dimercaptosuccinic acid	Thiol and Carboxyl	
Water fleas (Daphnia magna) (Exposure)	Fe_2_O_3_	33.3*	10–100 mg L^−1^	None (bare)	Hydroxyl	Significant toxicity, the ingestion and accumulation of coated and uncoated IONPs in the gastrointestinal tract [70]
		50.4*	1–100 mg L^−1^	Dimercaptosuccinic acid	Thiol and Carboxyl	

It is difficult to compare the in vivo effects of IONPs; because some studies don’t easily provide information of NPs; and in vivo interactions is more complex when compared with in vitro. Above studies proposed that Fe^3+^ is more toxic than Fe^2+^. IONPs reduced oxidative stress in drosophila and Parkinson model-rat, but in others were ineffective or toxic

^#^Parkinson model, * hydrate diameter

### IONPs may lead to iron accumulation

One of the main reasons to pinpoint IONPs applications is frequent implementation of IONPs to increase the contrast in MRI. For this purpose, the nanoparticles are guided to a target tissue by using an external magnetic field [9] which may intensify the levels of NPs and iron ions in the target tissue.

To investigate the effects of NPs on iron ion accumulation in the brain tissue, first it should be considered whether these NPs cross the BBB or not. The BBB is composed of endothelial cells, having tight junctions, which selectively permit the passage of some molecules and prevent the entrance of others [41]. Cengelli et al. have indicated that the uptake of IONPs by the brain endothelial cells of rats was low. They have proposed that the BBB damage in neurodegenerative disorders may facilitate the influx of IONPs to the brain [42]. In an agreement with this finding, Imam et al. recently reported that IONPs by producing ROS caused a damage to the membrane of rat’s brain endothelial cells [39]. Thus, IONPs are taken up slowly by endothelial cells or by destroying cellular membranes cross the BBB.

After crossing the BBB, it must be studied whether the neural system cells absorb IONPs or not. PC12 cell line is one of the cellular models for neural differentiation. In the presence of NGF, PC12 cells differentiate to dopaminergic neurons [43]. Pinkernelle et al. (2012) demonstrated the uptake of IONPs by PC12 cells, primary neurons of the cerebellum, and glial cells, including microglia, olygodendrocytes, astrocytes, and Schwann cells. Noticeably, their data approved the presence of NPs in the cells [44]. IONPs could disturb the cellular membrane of cortical neurons; the scanning electron microscopy pictures demonstrated that IONPs removed the cell membrane [45]. Thus, the nanoparticles can enter into the neural system cells and exert their functions.

The mechanism used by cells to uptake NPs may be phagocytosis, various type of endocytosis or diffusion [44]. Large IONPs are absorbed by endocytosis; but small IONPs incorporated into a cell by pinocytosis [46]. IONPs, after placing in the endosome, are degraded and released iron ions into the cytoplasm [47–49]. Volatron et al. simulated the medium of the endosome, with pH:4.7 and in presence of citrate, and showed degradation of IONPs in this medium. In absence of citrate, acidic pH could not break down IONPs [48]. Released iron ions introduce to physiological pathways; for example, they participate in hemoglobin synthesis, or is stored by ferritin in cells [47, 48]. IONPs affect iron hemostasis and cause upregulation of proteins related to storage/export of iron from a cell, or lead to downregulation of proteins related to iron uptake into the cell, such as TfR1 [47]. Laskar et al. proposed iron, released by IONPs degradation, induces an increased level of nuclear ferritin to reduce the toxic effect of excess iron on DNA. Furthermore, the cells which were exposed to IONPs, in order to export iron, incited ferroportin expression and ferritin secretion [49]. These studies indicate IONPs can influence iron pool in cells.

Iron accumulation in a cell depends on the initial concentration and properties of NPs, including size, shape, coating, and a functional group, and also cell type. Yu et al. have explained the concentration of iron in the cells increased depend on the dose of IONPs [50]. In addition, the dose of IONPs determines their longevity in a tissue. When IONPs were injected into a tissue in a high concentration, they were not completely removed from the tissue; even two months after injection; but NPs in a low concentration were deleted within three weeks [51]. Therefore, the high dose of IONPs causes the tissue cells to be exposed to IONPs for a long time.

Smaller NPs could accumulate in higher concentration then the larger ones in the cell [46]. A recent study has suggested that spherical nanoparticles compared with cubes had less contact surface for degradation; thus degraded more slowly [47]. Coating of IONPs with dextran has reduced degradation rate compared with citrate coating [48]. IONPs containing amine groups have positive charges, and also are taken up by a cell more than IONPs, which have negatively charged -carboxyl or hydroxyl groups [39]. Ligand bonded-IONPs can easily bind to their targeted cells, for example, folic acid bonded-IONPs bind to breast cancer cells [52]. Thus, the properties of IONPs affect their cellular uptake and degradation rate.

Cell type is an important factor to uptake IONPs. As explained above, cells such as microglia uptake a great content of IONPs [44]; but the brain endothelial cells absorb lower amount [39]. Any factor, which changes the uptake of IONPs or the degradation rate of them, can affect iron accumulation in a tissue and a cell; thus, these factors must be considered for applying IONPs in the CNS.

### IONPs may lead to oxidative stress

**Fig. 1:**
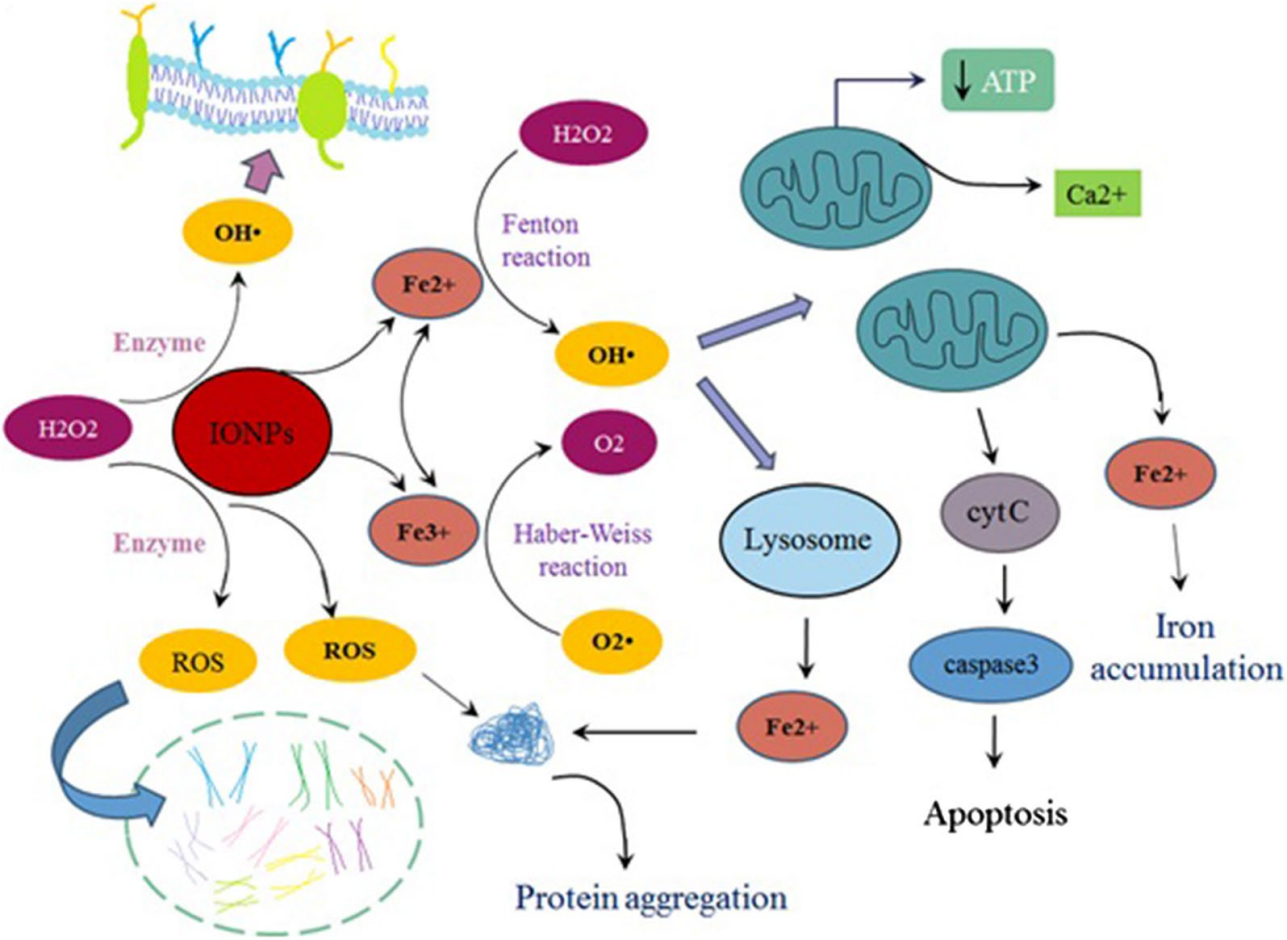
IONPs can cause iron accumulation, oxidative stress, and protein aggregation. IONPs present a large surface area for redox cycling [9]; in addition, the iron ions released from IONPs can also contribute to Fenton’s reaction, produce OH^·^ from H_2_O_2_, and finally lead to oxidative stress [57]. Reactive oxygen species (ROS) may directly damage DNA, cell membrane, and organelle’s membrane. ROS result in release of Ca^2+^ and cytochrome C from mitochondria, and therefore, induction of apoptosis [11]. Furthermore, ROS leads to the release of iron from lysosomes and mitochondria through damaging organelle’s membrane, [18], and iron accumulation in cytosol. Both ROS and free iron interact with a number of proteins, change their conformation, and mediate their aggregation [25]

Numerous studies have indicated the cytotoxicity of IONPs in vitro and in vivo [9, 17, 53–55]. In contrast, a number of studies have acclaimed that IONPs are non-toxic or even useful [40, 52, 56]. IONPs present a large surface area for redox cycling. In addition, separation of iron ions from their surface by enzymatic activity, may also produce ROS [9]. The iron ions released from IONPs can contribute to Fenton’s reaction and generate ROS from H_2_O_2_ and superoxide (Fig. 1) [57]. In contrary, Zhang et al. declared that bare Fe_3_O_4_ had a catalytic role and could reduce the effect of H_2_O_2_ on mouse fibroblastic cells [40]. Pongrac et al. discussed however neural stem cells incubated with IONPs showed a low level of ROS, a severe reduction was observed in GSH level of the cells. They also detected mitochondrial membrane hyperpolarization. Pongrac et al. have acclaimed oxidative stress has diminished mitochondrial membrane potential [58]. Thus, IONPs actually can activate the loop between reduced GSH, oxidative stress, and iron accumulation in neural stem cells.

Numerous factors determine the interaction of IONPs with cellular components, such as the oxidation state of iron, concentration, size, coating, and functional group. The oxidation state of iron (Fe^2+^ or Fe^3+^) in NPs is an important factor to determine NPs toxicity. Fe^3+^ in Fe_2_O_3_ is more toxic than Fe^2+^ in Fe_3_O_4_ and causes more DNA oxidation [59]. Fe_2_O_3_ has shown cytotoxicity in the most of the recent studies (see Table 1 and 2); for instance, Wu et al. reported Fe_2_O_3_ led to an increase in H_2_O_2_ content in PC12 cells [55].

The concentration of IONPs is a critical factor to determine their effects. The most of the studies investigated dose and time-dependent effects of IONPs; for example, Naqvi et al. reported that the viability of murine macrophages dose and time-dependently diminished by Fe_2_O_3_ [17]. Jarockyte et al. also, showed a dose and time-dependent reduction of viability by Fe_3_O_4_ in mouse embryonic fibroblasts (NIH3T3) [51]. Low doses of Fe_3_O_4_ declined oxidative stress in the stratum of Parkinson-model rats [60]. In contrast, high dose of IONPs induced oxidative stress in PC12 cells and resulted in altering the expression of mitochondrial enzymes, lipids peroxidation and cell membrane damage [9, 55].

The size of NPs may affect their cytotoxicity. After cellular uptake, small NPs degrade sooner than the large NPs. Large NPs, compared with the smalls, have more affinity to agglomerate; then they are easily taken up by macrophages [61]. Since smaller NPs present more reactive surface areas, therefore generate more ROS compared with the larger particles [59]. A recent study has demonstrated that 10 nm-NPs may lead to a significant increase in ROS level in endothelial cells [39]. In contrast, Yu et al. have explained that bare NPs, in 30 nm size, have incited ROS formation; but the bare NPs, in 5 nm size, have not altered ROS content [50]. The effects of size in toxicity of IONPs needs to more investigation.

The coating and the surface charge of IONP may influence its interaction with cellular components [59]. In study of Yang et al. coating by aminopropyltrimethoxysilane (APTMS) intensified the toxicity of bare NPs and led to dose-dependent diminished viability, membrane damage, and decreased the stability of DNA. The presence of amine groups on the NPs surface leads to more endocytosis of IONPs by cell and more concentration in the cell. The amine group has positive charged and more affinity to interact with negative charged DNA [46]. In contrast, in Yu et al. study bare NPs significantly increased ROS and cellular death, but coating of NPs by dextran or polyethylene glycol reduced ROS formation and cell toxicity [50]. Therefore, the type of coating is so effective in NPs toxicity.

Another factor in NPs toxicity is the type of target cells. As explained before, the uptake of IONPs by the different cells is different [44]. In addition, the interaction of normal and cancerous cell with NPs is different. The bare Fe_3_O_4_ diminished viability of normal dermal fibroblast, but promoted viability of fibrosarcoma in doses less than 800 µg mL^−1^ [46]. Thus, for depleting IONPs toxicity in cells, it is necessary to consider the concentration, size, shape, and coating of NPs, and also target cell type; and also, it seems vital using IONPs in combined with antioxidants.

### IONPs may lead to protein aggregation

As mentioned before, IONPs can lead to iron accumulation and oxidative stress. Given to the relationship between iron accumulation, oxidative stress, and protein aggregation, it is not surprising that IONPs can promote protein aggregation; as seen recently IONPs have caused an increase in αS expression in human neuroblastoma cells [39]. According to the role of oxidative stress in protein aggregation of αS [30] and Aβ [35], IONPs may result in protein aggregation. The factors such as charge, coating and side-groups influence IONPs function. Researchers showed that the presence of uncoated IONPs increased the formation of αS aggregation, while IONPs coated with lysine inhibited protein aggregation. It was proposed that uncoated IONPs resulted in promoting protein aggregation, by altering the ionic potential of soluble [28]. Studies indicated that the lower concentration of positively-charged IONPs may cause Aβ fibrillation compared with neutrally- or negatively-charged IONPs. Also, it has been proposed the presence of fluorinated, sulfated, or sulfonated groups on the surface of NPs can inhibit the formation of Aβ fibril [62]. Therefore, the application of suitable coatings and side-groups can reduce the damages of IONPs on the neural cells.

### IONPs may lead to apoptosis

A recent study has demonstrated that IONPs motivated long-term depolarization of neural stem cell membrane and hyperpolarization of mitochondrial membrane in the cell [58]. The inhibition of Na^+^–K^+^ ATPase transporter involved in maintenance of resting potential of cell membrane, has led to membrane depolarization [63], as observed in the brain of rats by Kumari and colleagues [64]. Depolarization of cellular membrane can cause Ca^2+^ ions entry into the cell and finally apoptosis through activation of *N*-methyl-d-aspartate (NMDA) receptors [63]. Wu et al. observed an increase in expression of *bax* and a decrease in expression of *bcl*-*2* in the PC12 cells exposed with IONPs [55]. Sarkar and Sil indicated that IONPs, in addition to upregulation of Bad and downregulation of Bcl-2, caused a reduction in the potential of mitochondrial membrane, the release of cytochrome c from mitochondria, and finally activation of caspase 3 in hepatocytes exposed to IONPs. They concluded IONPs could activate apoptosis through a mitochondria dependent way [65]. Therefore, IONPs may trigger apoptosis pathway in the cells.

## Discussion and conclusion

**Fig. 2:**
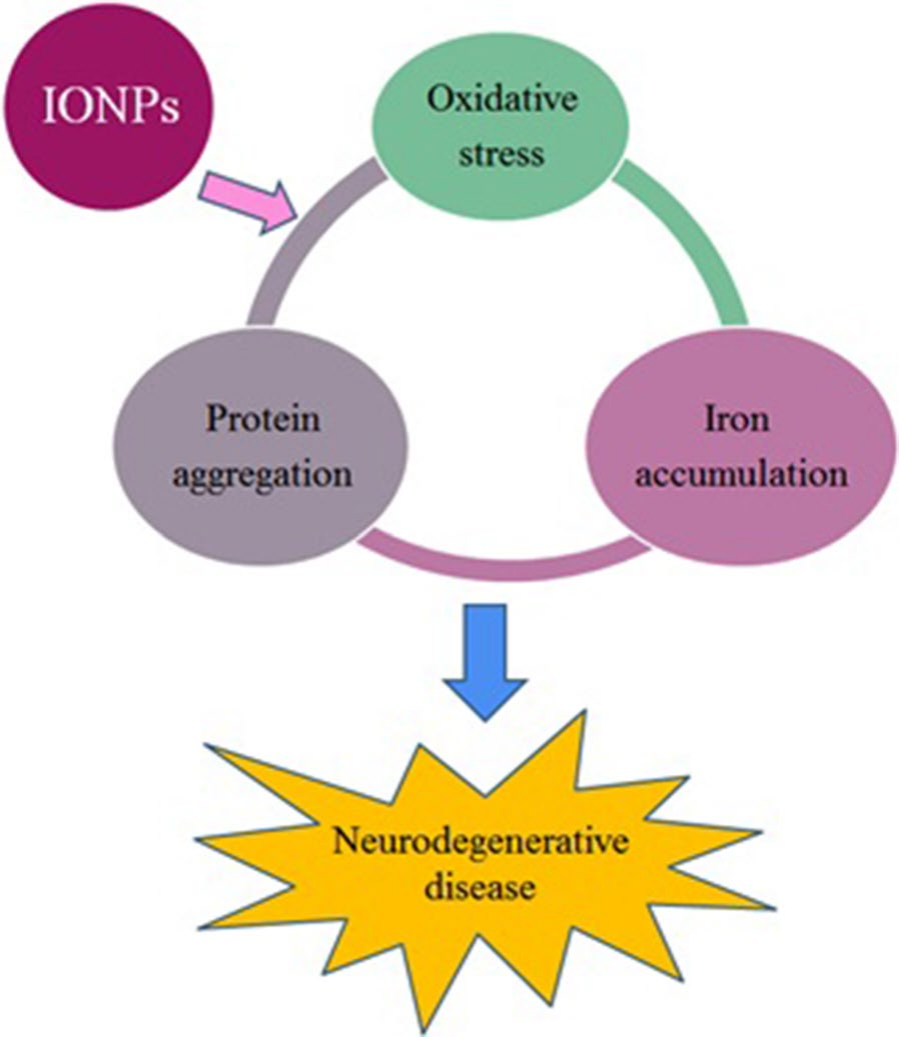
The summary of this study. Iron oxide nanoparticles (IONPs) can activate a positive feedback-loop between iron accumulation, oxidative stress, and protein aggregation and therefore, they may lead to neurodegenerative disease

As mentioned before, there is a positive feedback-loop between iron accumulation, oxidative stress, and protein aggregation in which one factor promotes other factors. IONPs can activate this loop by induction of iron accumulation, oxidative stress, or protein aggregation (Fig. 2). In addition, IONPs may provoke apoptotic cellular death in the neurons. Given the role of iron accumulation, oxidative stress, protein aggregation, and apoptosis in neurodegenerative diseases, IONPs may induce neurodegeneration.

But the toxicity of IONPs is affected by their properties, including size, shape, concentration, surface charge, the type of coating and functional groups. Therefore, in order to application of IONPs in the CNS, consideration of IONPs properties is so essential.

## Abbreviations

Aβ: amyloid beta
αS: α-sinculein
APP: amyloid precursor protein
APTMS: aminopropyltrimethoxysilane
APTS: aminopropyltriethoxysilane
BBB: blood brain barrier
CNS: central nervous system
DMT1: divalent metal transporter1
GSH: glutathione
GPx4: glutathione peroxidase4
6-OHDA: 6-hydroxydopamine
6-OHDA-Q: 6-hydroxydopamine quinone
IONPs: iron oxide nanoparticles
IRE: iron response element
IRP: iron response protein
MRI: magnetic resonance imaging
NMDA: *N*-methyl-d-aspartate
NP: nanoparticle
PEA: poly-(dimethylamine-coepichlorhydrin-co-ethylendiamine)
PEG: polyethylene glycol
PEI: polyethylenimine
ROS: reactive oxygen species
Sn: *Substantia nigra*
TEOS: tetraethylorthosilicate
TfR: transferrin receptor
